# *Sphingomonas* clade and functional distribution with simulated climate change

**DOI:** 10.1128/spectrum.00236-24

**Published:** 2024-04-04

**Authors:** Bahareh Sorouri, Nicholas C. Scales, Brandon S. Gaut, Steven D. Allison

**Affiliations:** 1 Department of Ecology and Evolutionary Biology, University of California, Irvine, California, USA; 2 Institute of Arctic Biology, University of Alaska Fairbanks, Fairbanks, Alaska, USA; 3 Department of Earth System Science, University of California, Irvine, California, USA; Connecticut Agricultural Experiment Station, New Haven, Connecticut, USA

**Keywords:** *Sphingomonas*, metagenomics, climate gradient, traits, phylogenetics

## Abstract

Microbes are essential for the functioning of all ecosystems, and as global warming and anthropogenic pollution threaten ecosystems, it is critical to understand how microbes respond to these changes. We investigated the climate response of *Sphingomonas*, a widespread gram-negative bacterial genus, during an 18-month microbial community reciprocal transplant experiment across a Southern California climate gradient. We hypothesized that after 18 months, the transplanted *Sphingomonas* clade and functional composition would correspond with site conditions and reflect the *Sphingomonas* composition of native communities. We extracted *Sphingomonas* sequences from metagenomic data across the gradient and assessed their clade and functional composition. Representatives of at least 12 major *Sphingomonas* clades were found at varying relative abundances along the climate gradient, and transplanted *Sphingomonas* clade composition shifted after 18 months. Site had a significant effect (PERMANOVA; *P* < 0.001) on the distribution of both *Sphingomonas* functional (R^2^ = 0.465) and clade composition (R^2^ = 0.400), suggesting that *Sphingomonas* composition depends on climate parameters. Additionally, for both *Sphingomonas* clade and functional composition, ordinations revealed that the transplanted communities shifted closer to the native *Sphingomonas* composition of the grassland site compared with the site they were transplanted into. Overall, our results indicate that climate and substrate collectively determine *Sphingomonas* clade and functional composition.

**IMPORTANCE:**

*Sphingomonas* is the most abundant gram-negative bacterial genus in litter-degrading microbial communities of desert, grassland, shrubland, and forest ecosystems in Southern California. We aimed to determine whether *Sphingomonas* responds to climate change in the same way as gram-positive bacteria and whole bacterial communities in these ecosystems. Within *Sphingomonas*, both clade composition and functional genes shifted in response to climate and litter chemistry, supporting the idea that bacteria respond similarly to climate at different scales of genetic variation. This understanding of how microbes respond to perturbation across scales may aid in future predictions of microbial responses to climate change.

## INTRODUCTION

Microorganisms are critical for ecosystem functioning and are threatened by the anthropogenic effects of climate change (1, 2). Furthermore, microbial communities drive planetary biogeochemical cycles—such as carbon and nitrogen fluxes—that all organisms require for survival (3). Therefore, it is important to understand the implications of climate change for microbial composition and functioning. However, due to high microbial abundance and diversity, it is difficult to predict how microbial communities will collectively respond to environmental shifts (4). Moreover, the ecological niche of a single microbial strain—or a genetic variant of a species—can vary depending on the geographic origin of the strain or prior exposure to stress, which further complicates the predictions (5, 6).

When investigating microbial response to climate change, it is important to consider the scale of genetic variation. For example, the response to climate change varies across domains, as well as broad clades within bacteria and fungi (7–10). Additionally, differential responses to climate occur not only at community scales (11–13) but also at finer scales. Microdiversity refers to the genetic variation within operational taxonomic units that have high genomic similarity (14–16). Microdiversity may also respond to global change—for instance, within the gram-positive bacterial genus *Curtobacterium*, taxa differentially adapted to local climates and shifted in abundance after transplantation (17). Furthermore, microdiversity within bacterial taxa is beneficial for ensuring the stability of microbial communities when the environment changes (18). Although many studies have compared microbial responses to changes across genera, it is important to consider the microdiversity within a genus, since it influences microbial niches, traits, and biogeography (19).

Changes in microdiversity may be reflected in functional traits (13, 20, 21). Therefore, analysis of genomes and ecologically relevant functional traits could help predict the ecosystem implications of environmental change (13, 22–24). For example, gene-based functional groups in soil bacteria vary in their tolerance to perturbation, such as high salinity (25, 26). Analysis of functional traits can also be used to identify life history strategies that are based on an organism’s phenotypic characteristics. One such example is the Y-A-S trait-based framework for microbial growth Yield, resource Acquisition, and Stress tolerance (27).

Building on the Chase et al. (17) study of *Curtobacterium*, we aimed to test how microdiversity responds to climate change in *Sphingomonas*, another key bacterial clade. We leveraged the same field experiment used for the *Curtobacterium* study in which microbial communities were reciprocally transplanted across a Southern California climate gradient with temperature and precipitation varying inversely across five sites: desert, scrubland, grassland, pine-oak, and subalpine (7, 28). *Sphingomonas* is the most abundant gram-negative bacterial genus found in these climate gradient sites (20). Along with *Curtobacterium*, *Sphingomonas* contributes to the ecosystem process of litter decomposition. *Sphingomonas* can also depolymerize lignin, a chemical-resistant plant structural polymer (29, 30), and catalyze the bioremediation of chemically contaminated soils by degrading complex polycyclic aromatic hydrocarbons (31). In addition, some *Sphingomonas* can improve plant growth under stressful salinity, drought, and heavy metal conditions (32).

**Fig 1:**
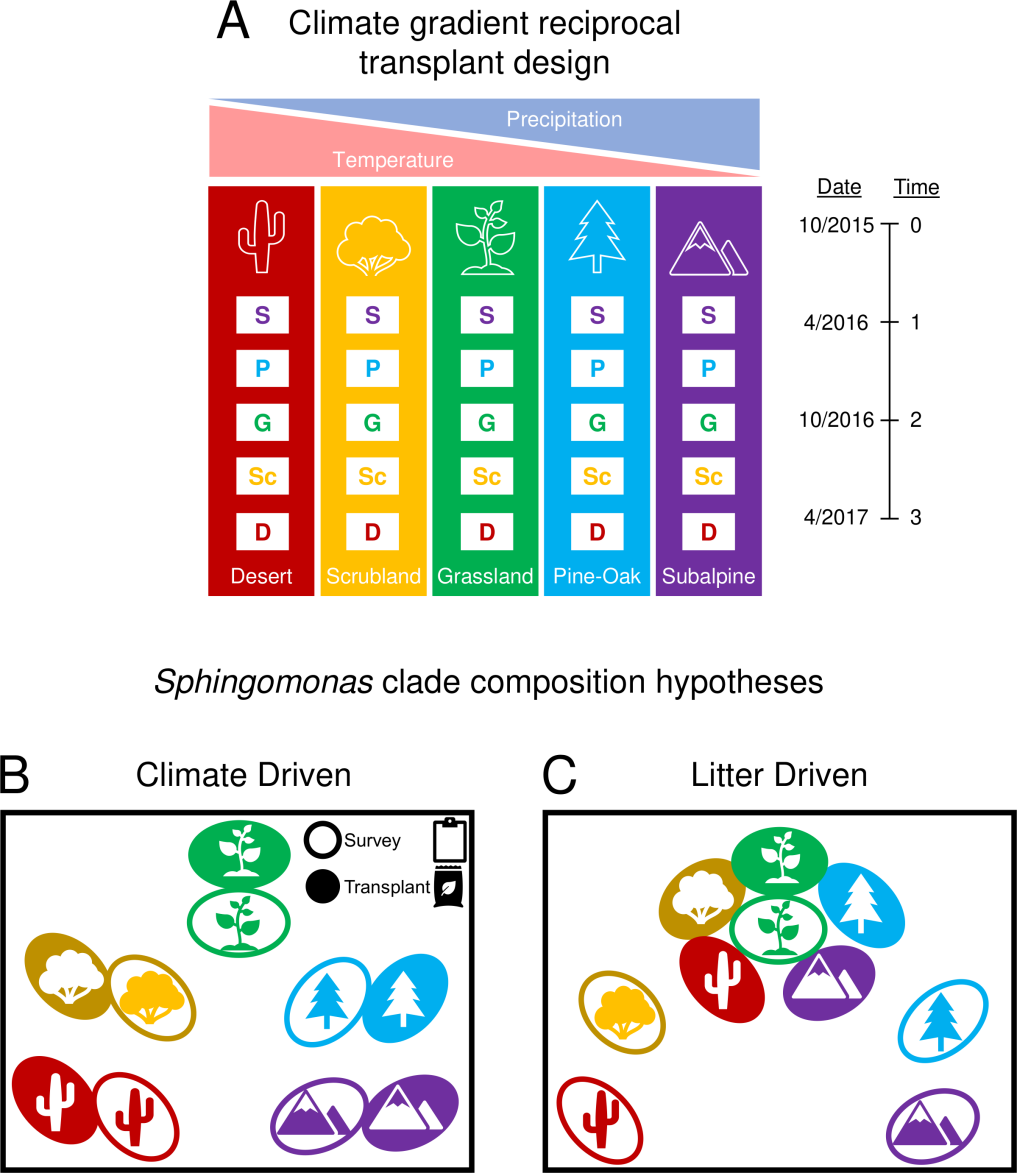
Microbial community reciprocal transplant design and hypotheses driving *Sphingomonas* clade composition after 18 months in the field. (A) Schematic of reciprocal transplant experiment across a climate gradient where temperature and precipitation vary inversely. The sites include the desert (D), scrubland (Sc), grassland (G), pine-oak (P), and subalpine (S) ecosystems. All colors and icons remain consistent across the figures. We hypothesized that (B) site environment (e.g., climate) determines *Sphingomonas* composition after 18 months. Thus, the composition within bag transplant and survey samples will be similar at each site. (C) Alternatively, since all microbial communities within transplants were inoculated onto grassland litter, the grassland substrate might drive *Sphingomonas* composition, causing transplants to converge on the grassland survey samples.

As in prior studies of *Curtobacterium* (17, 20) and whole microbial communities (7), we investigated the phylogenetic and functional diversity of *Sphingomonas* across the climate gradient. Using shotgun metagenomic data, we tested how the composition and functional potential of the *Sphingomonas* genus shifted in response to climate change as simulated in the reciprocal transplant experiment (Fig. 1A). Based on the results with *Curtobacterium* (17), we hypothesized that the clade and functional composition in transplanted communities would correspond to the site environment and climate (Fig. 1B). However, all transplanted microbial communities were inoculated onto a common grassland litter; hence, we alternatively hypothesized that the *Sphingomonas* clade and functional composition might converge due to environmental selection by litter type (Fig. 1C). Substrate chemistry is a potentially important control on microbial community assembly that can also shift with climate change (33, 34); therefore, we aimed to assess both climate and substrate drivers of *Sphingomonas* composition in the transplant experiment. Overall, our goal was to evaluate the consistency of microbial response to climate change at multiple phylogenetic levels.

## MATERIALS AND METHODS

### Reciprocal transplant design and metagenomic samples

We analyzed the metagenomic data from an 18-month reciprocal transplant across a Southern California climate gradient, as previously described in the study of Glassman et al. (7). Briefly, the climate gradient consists of five sites (desert, scrubland, grassland, pine-oak, and subalpine) across which temperature and precipitation vary inversely. Leaf litter was collected from each site on September 11, 2015. Subsequently, the leaf litter was homogenized with coffee grinders and used to inoculate irradiated grassland leaf litter in sterilized nylon bags. The nylon litter bags had 0.22 µm pores (cat. No. SPEC17970; Tisch Scientific, Cleves, OH, USA) such that nutrients and water could move freely in and out of the bags, but bacteria and fungi could not.

On October 19, 2015, the transplant bags were placed in the five sites and destructively sampled after 6, 12, and 18 months. In total, 300 bags (five sites × five inoculums × four replicates × three time points) were deployed, and 100 bags were collected at each time point. Additionally, at each time point, survey samples comprising native microbial communities on their native litter were collected adjacent to each plot. Time point 0 (T0) refers to the time that all litter bags were deployed into the field. Time point 1 (T1) corresponds to the sampling at 6 months, time point 2 (T2) at 12 months, and time point 3 (T3) at 18 months.

We analyzed metagenomic data from climate gradient samples sequenced previously (35). Briefly, DNA was extracted from 0.05 g of ground leaf litter using the FastDNA SPIN Kit for Soil (Mo Bio). The DNA was subsequently cleaned with the Genomic DNA Clean and Concentrator kit (Zymo Research). Clean samples were diluted, processed with the Nextera XT library Prep kit, and sequenced with the Illumina HiSeq4000 instrument with 150 bp paired-end reads. Raw metagenomic data were found on the metagenomic analysis server (Metagenomic Rapid Annotations using Subsystems Technology, MG-RAST) under project ID mgp17355 (36). The data consisted of initial T0 metagenomes (*N* = 20), 18-month T3 survey metagenomes (*N* = 20), and 18-month T3 transplant metagenomes (*N* = 99). There was one missing replicate from the desert inoculum in the grassland site from the 18-month transplant samples. Metagenomic data were not available for the transplant bags at T1 and T2; therefore, we compared the T0 initial data with both the 18-month T3 survey and transplant data.

### Sphingomonas identification from metagenomic samples

We trimmed and quality-filtered the metagenomic data using trimmomatic v0.36 (37). We used bwa v0.7.17 and samtools v1.10 (38, 39) to filter out plant and fungal sequences (17). For our analyses, we used the forward reads only to simplify read counting.

Using previously published results, we investigated 252 high-quality, publicly available *Sphingomonas* genomes that comprised a phylogenetic tree with 12 clades (40). Of the previously identified 444 shared core genes within the *Sphingomonas* genomes (40), we selected 23 marker core genes (Table S1) that also appeared in a reference genomic amino acid database developed by Chase et al. (35). We appended the 23 core genes from each *Sphingomonas* genome to the Chase et al. (35) reference genomic database, which together served as our reference database for DIAMOND v2.0.4.142 BLASTX (41). We included the genes from the reference genomic database because we wanted to extract only *Sphingomonas* core gene hits and avoid matching metagenomic sequences that were not *Sphingomonas*. Forward FASTA reads from metagenomic samples were queried against the protein reference database with default Basic Local Alignment Search Tool X (BLASTX) parameters (41). Using a reciprocal BLAST against known sequences, we determined a conservative threshold of a percent identity value of at least 98% and an E-value of less than 1e^−20^ to be sure that the hits belong to *Sphingomonas*. All relevant data can be found on GitHub, https://github.com/baharehsorouri/sphing_climategradient.

When querying the metagenomic sequences, some matched to only one clade, whereas others matched to multiple clades. Those that hit only one clade were assigned to that clade. For the sequences that hit multiple clades, we first checked whether all hits to one of the clades showed consistently higher percent identity (e.g., 100%); if so, the sequence was assigned to that clade. If not, we assigned the sequence to a “pseudo clade” consisting of all clades that matched the query sequence with the same, highest identity. For example, if a query sequence matched five reference sequences from clade 1 with 100% identity, four reference sequences from clade 2 with 100% identity, and three reference sequences from clade 3 with 98% identity, then the query was assigned to pseudo clade 1–2. Because there are hundreds of potential pseudo clades, we grouped the sequences assigned to rarer pseudo clades into a single broad pseudo clade for subsequent analyses. Pseudo clades that had less than 10 hits were combined into the broad pseudo clade.

**Fig 2:**
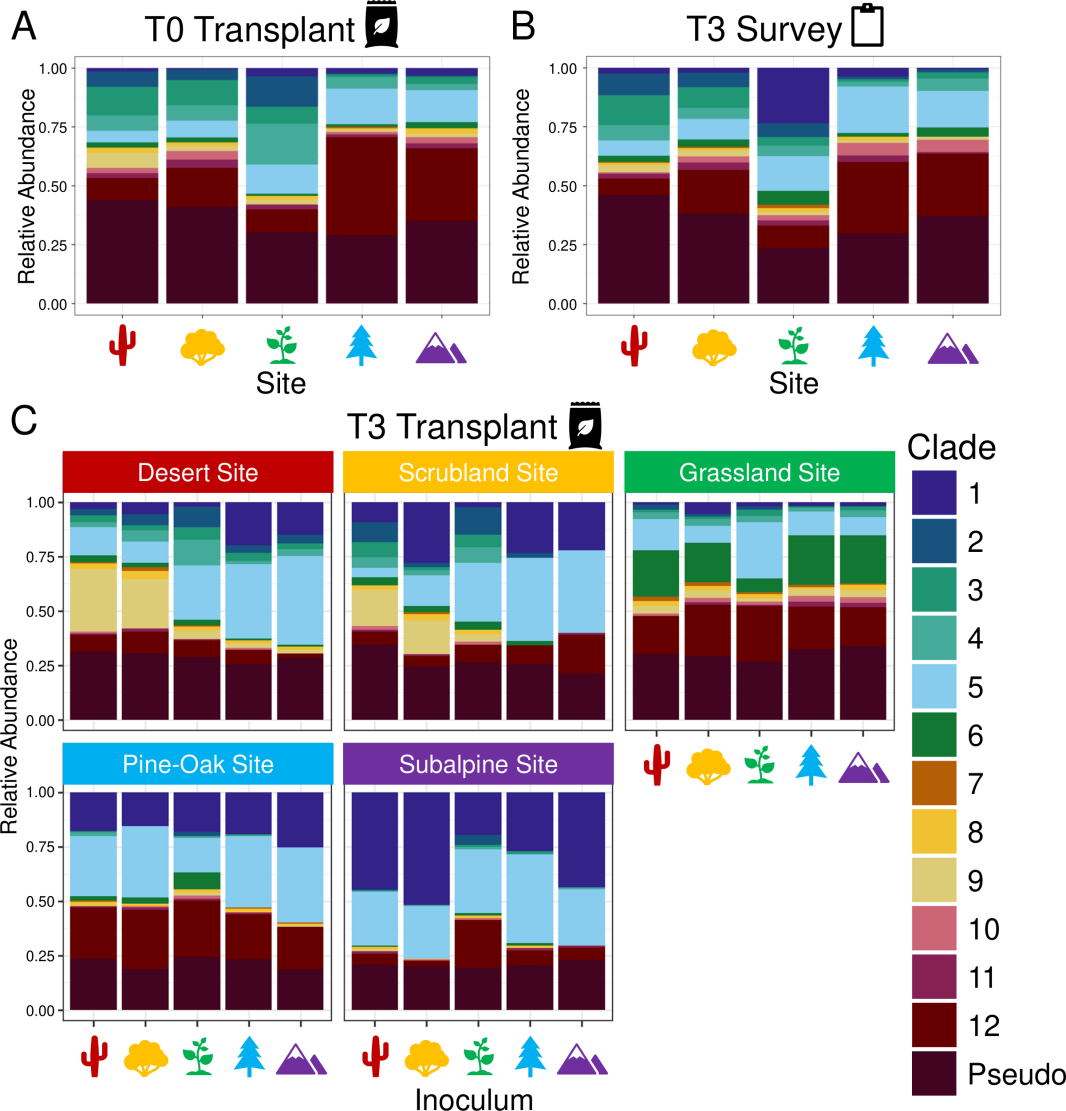
*Sphingomonas* clade distribution across the climate gradient in (A) T0 transplant samples, (B) T3 survey samples, and (C) T3 transplant samples. For (A) and (B), the clade relative abundances are represented for each site. For the T3 transplant, the facet boxes are labeled and colored by the site, and the x-axis indicates inoculum within each site. Facet and icon colors reflect the site, whereas relative abundances are colored by clade. The pseudo clade category encompasses all pseudo clades.

For each of the climate gradient samples, we reported the total number of *Sphingomonas* sequences by clade. To account for differences in sequencing depth between samples, we rarefied the data to 50 with the EcolUtils v0.1 R package (42). We calculated the clade relative abundances by dividing by the total for each sample and visualized clade relative abundances using the ggpubr v0.4.0 R package (43). When visualizing clade relative abundances, we grouped all the pseudo clades into one larger category for simplicity (Fig. 2). One T3 desert survey sample and one grassland sample were removed during the rarefaction step due to low sequence coverage. Additional samples were also removed from the T3 transplant bags during rarefaction from the following sites: one desert, two grassland, two pine-oak, seven scrubland, and three subalpine. We performed a principal coordinate analysis (PCoA) on the clade relative abundance data using Bray-Curtis dissimilarity to visualize compositional differences between samples. A permutational multivariate analysis of variance (PERMANOVA) is a distance-based method that tests whether microbial composition is associated with the covariates (44); in our case, it was used to determine site and inoculum effect sizes from Bray-Curtis dissimilarity matrices. Due to differences in time point sample sizes, we ran separate PERMANOVAs for each time point. We also ran a permutational analysis of dispersion (PERMDISP) to determine the dispersion of individual samples within each group (45). All statistical analyses and data visualizations across the climate gradient were done in R v4.1.0; to ensure reproducible results from statistics using permutations, we set the seed value to 1 (46). The PCoA, PERMANOVA, and PERMDISP analyses were performed with the vegan v2.5–7 R package, and the PCoA was visualized with ggplot2 v3.4.2 (47, 48). Ellipses were drawn using the “stat_ellipse” function of ggplot2, whereas ellipses for groups with three replicates were visualized with the geom_mark_ellipse function of the ggforce v0.4.1 package (47, 49).

For the ordination, we calculated the median point for each axis within each site to calculate the T3 transplant and survey centroids. Additionally, we used the mean distances between centroids to determine which hypothesis was supported. To test the hypothesis that climate is responsible for convergence, we calculated the average distance (and standard deviation) between the T3 transplant centroids and their corresponding T3 survey centroid within the same site. To test the alternative hypothesis that grassland litter is responsible for convergence, we calculated the average distance between the T3 transplant centroids and the T3 grassland survey centroid.

### Sphingomonas functional genes

Again, using the previously described results, we investigated a subset of genome-based functional traits from the publicly available *Sphingomonas* genomes that were assigned to a Y-A-S life history category depending on their role in growth yield (Y), resource acquisition (A), or stress tolerance (S) (27, 40). Briefly, the Kyoto Encyclopedia of Genes and Genomes (KEGG) and Carbohydrate-Active enZymes (CAZy) databases were used to identify the genome-based functional traits using GhostKOALA v2.2. and dbCAN2 tools, respectively (50–53). In summary, the three genome-based traits associated with the Y strategy included amino acid-related enzymes, lipid biosynthesis proteins, and lipopolysaccharide biosynthesis proteins. CAZymes and polycyclic aromatic hydrocarbon degradation indicated the A strategy. Chaperones and folding catalysts, peptidoglycan biosynthesis and degradation proteins, and prokaryotic defense system proteins were the three genome-based traits attributed to the S strategy. We calculated the relative abundances of each of these eight trait categories for each of the 12 *Sphingomonas* clades designated by the publicly available genomes (40).

To infer the distribution of the eight genome-based traits across the climate gradient, we multiplied the trait relative abundances from the publicly available genomes by the clade relative abundances for the transplant and survey samples at 18 months. For this analysis, we recalculated the clade relative abundances across the climate gradient for just the 12 clades and removed the pseudo clade categories. We averaged the values by sample and used them to construct a matrix with rows corresponding to samples and columns corresponding to traits. PERMANOVA analyses were done on this matrix using the same methods described earlier for *Sphingomonas* clade relative abundances. Furthermore, we performed principal component analyses (PCA) using the “prcomp” function of the base R stats package (46). PCA summary statistics were used to calculate the factor loadings of the genome-based traits, meaning the correlation between the principal components and the underlying genome-based traits. The PCAs and factor loadings were visualized with ggplot 2 (47). The centroid calculations were done using the same methods described previously for clades.

## RESULTS

### Sphingomonas clade composition

We aimed to evaluate the representation of *Sphingomonas* clades (40) in metagenomic sequences across the climate gradient. Of 49,044 sequences identified as *Sphingomonas*, 34,003 matched to one of the 12 phylogenetic clades, and the remaining 15,041 were assigned to pseudo clades. Each of the 12 main clades was found across the climate gradient in both transplant and survey samples at varying abundance. *Sphingomonas* composition was similar in the initial inoculum at T0 and the survey samples at T3, indicating temporal consistency in clade composition of the native litter *Sphingomonas* (Fig. 2A and B). However, the grassland site had a higher relative abundance of clade 1 in the T3 survey samples compared with T0. When comparing the survey and transplant samples at 18 months, there was a distinct difference in the distribution of *Sphingomonas* clades (Fig. 2B and C).

Environmental conditions at the sites influenced the composition of *Sphingomonas* clades. Although both site and inoculum had significant (*P* < 0.001) effects on the distribution of transplanted *Sphingomonas*, site was the strongest predictor of composition with an R^2^ value of 0.400, whereas inoculum had an R^2^ value of 0.093 (Table 1). The strong site effect suggests that climate or other site conditions influence *Sphingomonas* composition following transplantation. There was also a significant (*P* < 0.001) site by inoculum interaction (R^2^ = 0.179), which had a stronger effect on *Sphingomonas* composition than inoculum. This result indicates that the inoculum effect varies by site (Fig. 2C).

**TABLE 1:** PERMANOVA statistics for factors explaining the clade composition of *Sphingomonas* within T3 transplanted samples

	Df	SS	R^2^	F	*P* value
Site	4	4.77	0.400	18.0	< 0.001
Inoculum	4	1.11	0.093	4.16	< 0.001
Site:Inoculum	16	2.13	0.179	2.01	<0.001

**Fig 3:**
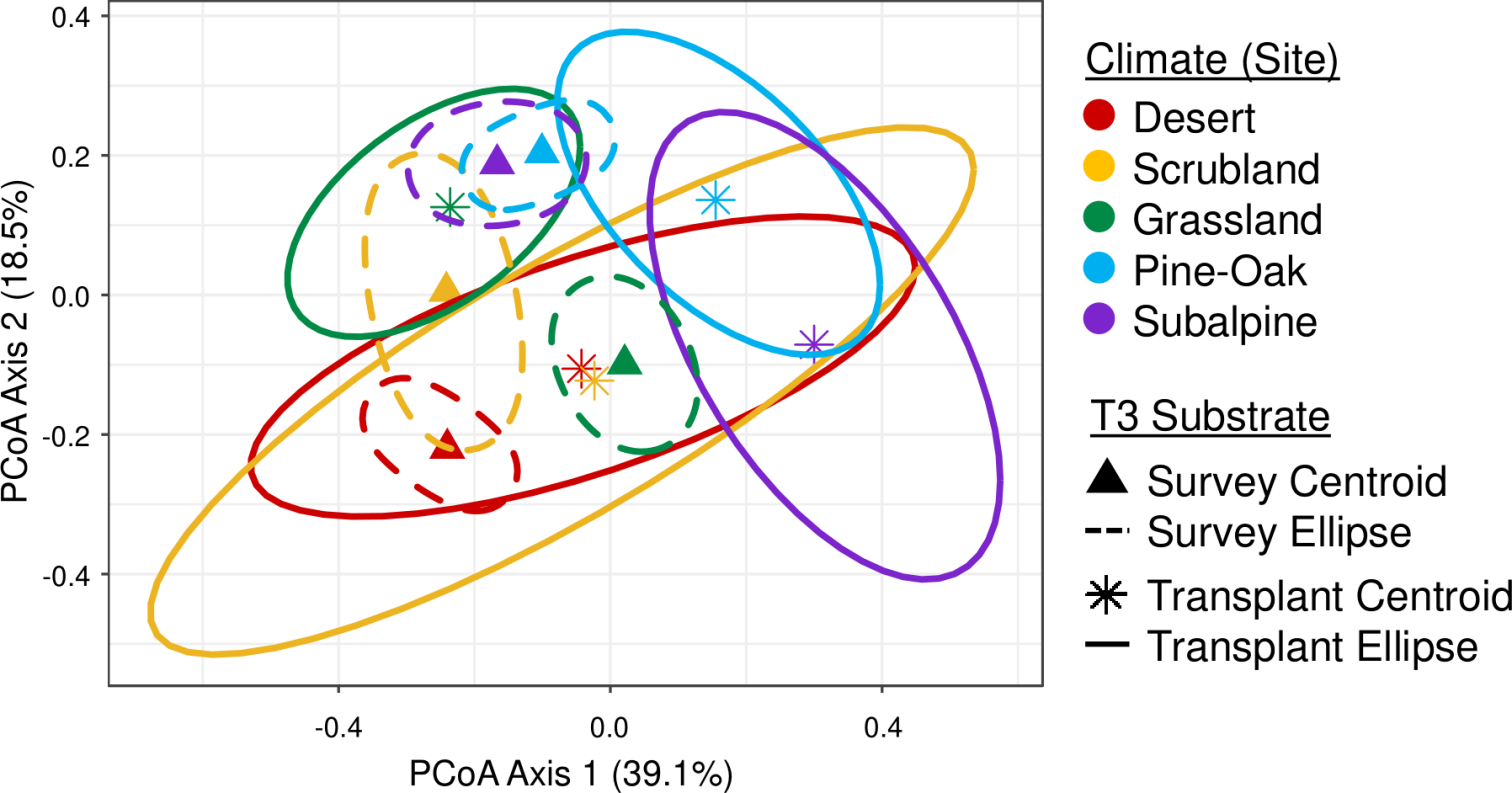
Principal coordinate analysis of *Sphingomonas* clade relative abundances within survey and transplant samples after 18 months. The colors reflect the sites, and the ordination was calculated with Bray-Curtis dissimilarity distances. Triangles represent survey centroids, and asterisks represent the centroids of transplant samples. Dashed-lined ellipses with a 95% CI encompass survey points, whereas solid-lined ellipses encompass the transplanted points.

Furthermore, the *Sphingomonas* clade composition within the grassland was similar across inocula, since the T3 transplanted samples at the grassland site had the tightest clustering in the PCoA (Fig. 3; Fig. S2). For the other sites, there was more variation in *Sphingomonas* clade composition across inocula in the transplanted bags compared with the survey bags (Fig. 3; Fig. S2). In the desert site, the relative abundances of clades 1, 2, 5, and 9 varied most across transplanted inocula. Within the scrubland site, relative abundances of clades 1, 2, 5, 9, and 12 varied most, whereas that of clades 1, 5, and 12 varied most in the subalpine site (Fig. 2C). Clade 7 was absent in most T3 transplant samples, and in the higher elevation pine-oak and subalpine sites, clades 1, 5, and 12 were the most abundant. The grassland site had the highest abundance of clade 6 within the transplanted bags (Fig. 2C). Overall, clades 1 and 5 played an important role in driving the site by inoculum interaction. Clades 1 and 5 varied widely across the transplanted inocula within the desert, scrubland, and subalpine sites. However, clade 1 was rare within the grassland site and consistent across inocula within the pine-oak site.

If *Sphingomonas* composition is determined primarily by litter substrate, we would expect transplanted communities to converge on the grassland survey community (Fig. 1C). The T3 survey samples not only clustered together by site but also partially converged on the grassland survey community (Fig. 3). The centroids of the *Sphingomonas* communities transplanted into the desert and scrubland were the closest to the grassland survey. The pine-oak, subalpine, and grassland transplants were further away from the grassland survey. Overall, the average distance between the centroids of the transplant samples and the grassland survey (0.201; SD = 0.134) was smaller than the average distance between the transplant centroids and survey samples within the same site (0.324; SD = 0.125). Therefore, there was also support for the hypothesis that *Sphingomonas* clades converged on the grassland litter. Although the transplant centroids converged, significant PERMDISP results (*P* < 0.001; R^2^ = 0.567; SS = 0.343; Df = 29) indicated that samples within groups differed in their dispersion or the spread within a site. Thus, because there was dispersion in the data, individual samples did not all converge on the grassland survey.

### Sphingomonas functional composition

**Fig 4:**
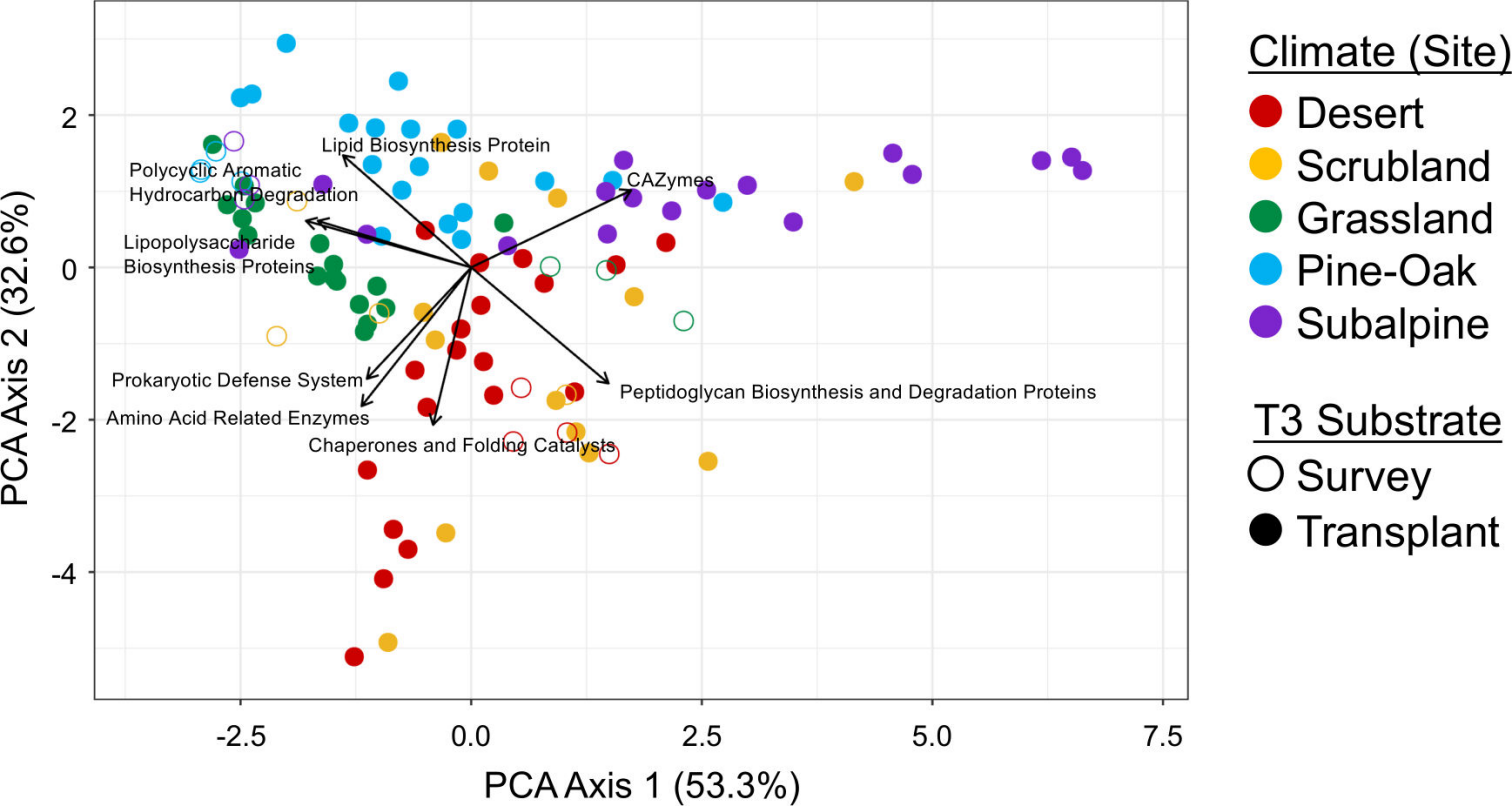
Principal component analysis of the predicted *Sphingomonas* genome-based YAS functional genes within the survey and transplanted samples after 18 months. The ordination does not include trait information from pseudo clades. Colors reflect sites across the climate gradient, open symbols indicate survey samples, and closed symbols represent transplanted samples. Amino acid-related enzymes, lipid biosynthesis proteins, and lipopolysaccharide biosynthesis proteins represent the growth yield (Y) life history strategy. CAZymes and polycyclic aromatic hydrocarbon degradation reflect resource acquisition (A). Chaperones and folding catalysts, prokaryotic defense system, and peptidoglycan biosynthesis and degradation proteins indicate the stress tolerance (S) life history strategy.

Since *Sphingomonas* functional gene content reflects habitat preferences (40), we predicted and investigated the distribution of eight *Sphingomonas* genome-based functional traits to determine whether they supported our hypotheses (Fig. 1B and C). Similar to the clade composition patterns, site and inoculum had significant (*P* < 0.001; *P* < 0.05) effects on *Sphingomonas* functional composition in transplants by T3; however, the site by inoculum interaction was not significant (Table 2; Fig. 4; Fig. S3). As with the clade composition, site had the strongest effects on the *Sphingomonas* functional gene distribution (R^2^ = 0.465, *P* < 0.001), whereas inoculum had a weaker effect (R^2^ = 0.058, *P* < 0.05). The patterns of convergence on the grassland survey for functional traits were nearly identical to the patterns for clade composition in that the average distance between T3 bag centroids and the T3 grassland survey centroid (2.10; SD = 0.855) was smaller than the average distance between the T3 bag centroids and their respective T3 survey samples (2.67; SD = 1.47) from the same site (Fig. S3).

**TABLE 2:** PERMANOVA statistics for factors explaining the predicted functional composition of *Sphingomonas* within T3 transplants

	Df	SS	R2	F	*P* value
Site	4	5.58E-03	0.465	19.1	< 0.001
Inoculum	4	7.18E-04	0.0575	2.36	0.027
Site:Inoculum	16	1.48E-03	0.118	1.21	0.247

To infer the preferred life history strategies of the microbial communities across the climate gradient, we also investigated the factor loadings of the genome-based traits on the PCA distribution. The subalpine site is associated with high CAZyme abundance, suggesting environmental selection for the resource acquisition life history strategy (Fig. 4; Fig. S4). In contrast, although the grassland and pine-oak sites appear to select for polycyclic aromatic hydrocarbon degradation, they also select for the growth yield life history strategy and are more closely associated with lipopolysaccharide biosynthesis proteins and lipid biosynthesis proteins, respectively. The desert and scrubland sites are occasionally associated with CAZymes but selected mainly for the stress response life history strategy based on associations with peptidoglycan biosynthesis proteins, and chaperones and folding catalysts (Fig. 4; Fig. S4).

## DISCUSSION

Our results suggest that microbial responses to environmental change are consistent across bacterial clades at the genus level. The *Sphingomonas* clade composition varies across the climate gradient, indicating that clades may be differentially adapted to site conditions, including climate. Furthermore, *Sphingomonas* clade composition shifted within the genus over an 18-month reciprocal transplant (Fig. 2). These findings thus support the hypothesis that site climate drives *Sphingomonas* clade and trait composition (Fig. 1B and 3; Fig. S3; Table 1). Although factors such as nutrient availability and vegetation cover also vary across the sites, climate was likely the site factor with the greatest impact on bacteria in the transplanted litter bags (7). At the same time, there was support for our alternative hypothesis that the grassland substrate was responsible for clade and trait convergence (Fig. 1C and 3; Fig. S3), suggesting that indirect effects of climate change via plant community shifts may also influence *Sphingomonas* response to climate.

The outcome that site and abiotic climate conditions drive clade and functional compositional shifts is in line with previous findings globally (54) and across this Southern California climate gradient. Glassman et al. (7) and Chase et al. (17) found that climate drives distinct bacterial composition at strain to community levels. Our study shows that these results extend to the *Sphingomonas* genus, an abundant gram-negative bacterial clade (Fig. 3; Table 1). Although some climate response traits are deeply conserved (24, 55, 56), climate response also occurs at fine scales, as our results suggest for *Sphingomonas* and prior research shows for *Prochlorococcus* in the ocean (13). Therefore, responses to climate change can occur across different scales of genetic variation.

The patterns in the distribution of *Sphingomonas* clades and functional genes across the climate gradient to some extent reflect the climate conditions of the sites (Fig. 4). Within both survey and transplant samples at T3, oftentimes, the desert and scrubland sites grouped together, as did the pine-oak and subalpine sites. These groupings may reflect similarities with respect to temperature, precipitation, and litter chemistry (28).

Although site conditions are a strong predictor of shifts in *Sphingomonas* clade composition (Fig. 3; Table 1), there was variation among the transplanted communities within each site after 18 months despite inoculation onto the same grassland litter (Fig. 2). It is likely that the different initial communities had varying levels of resilience to environmental changes, and microbial legacy effects from previous historical events may have prevented complete convergence (7, 17, 57–59). Additionally, *Sphingomonas* clades 1, 5, and 6 contain taxa that are found at high relative abundances in both plant and environmental habitats (40). These clades shifted the most in the T3 transplant after 18 months (Fig. 2). Perhaps, these clades are more abundant in the T3 transplant because they are better suited to survive on grassland litter across the climate gradient. Some species of *Sphingomonas* have symbiotic relationships with plants that improve plant growth and drought tolerance (60).

We were surprised to find that for both clade and functional composition, the survey and transplant samples within the grassland site did not closely converge and in fact were dissimilar (Fig. 3 and 4; Fig. S2 and S3). After 18 months, the grassland survey was more similar to the scrubland and desert transplant samples than to the grassland transplant samples. Given that the initial *Sphingomonas* clade composition of the grassland inoculum was similar to the grassland survey and they were both on grassland litter, we anticipated that the grassland survey and transplant samples would converge after 18 months (Fig. 1). It is possible that there were microclimate differences across bags that prevented convergence, although it is not clear why these differences would be more pronounced in the grassland. Alternatively, grassland substrates may vary from year to year, and microbial succession or ecological drift may have further contributed to the variation between the two groups (61). The combination of both litter substrate and bag microclimate may explain distinct *Sphingomonas* composition in grassland survey and transplant samples, given that the response of bacterial microdiversity to environmental perturbations is substrate- and ecosystem-dependent (21).

Trait-based approaches are useful for predicting and interpreting microbial responses to climate change. Trait responses to climate change simulated by transplantation closely followed clade responses, which is consistent with the trait variation across clades (40). For example, clade 1 had the highest abundance of CAZymes and was responsible for driving the differences in *Sphingomonas* clade and functional composition (40). We also observed that clade 7 was rare in our litter metagenomes, consistent with this clade’s dominance by clinical strains that may not possess the traits to live in a surface soil environment. Still, across the climate gradient, differences in the relative abundance of other environmentally prevalent clades such as 5 and 6 were not clearly related to the genome-based traits we measured. We recognize that there are likely finer-scale differences between traits, such as individual glycoside hydrolases (62), that we did not assess here. We also found a preference for specific life history strategies across the climate gradient from the genome-based functional traits (Fig. 4). The hottest, driest ecosystems along the climate gradient (desert and scrubland) selected for clades with the stress tolerance life history strategy, as supported by previous studies (27, 63, 64). Grassland and pine-oak ecosystems favored clades with the growth yield life history strategy. The cooler, wetter subalpine ecosystem selected more for resource acquisition strategies. Additionally, there may be functional differences in other ecologically relevant traits that we did not analyze.

We investigated the distribution of *Sphingomonas* clades and functional potential across a Southern California climate gradient. We found that the clade and functional composition shifted during an 18-month reciprocal transplant. Our findings indicate that the gram-negative *Sphingomonas* genus had compositional and functional responses similar to gram-positive *Curtobacterium* (17) and whole microbial communities (7). Collectively, these studies suggest that compositional and functional responses to climate change occur at various genetic scales, ranging from within *Curtobacterium* strains to within *Sphingomonas* clades and across clades within microbial communities. Understanding how microbes respond to perturbation at all these genetic scales may aid in future predictions of microbial responses to climate change.

## Data Availability

Raw metagenomic data were found on the metagenomic analysis server (MG-RAST) under project ID mgp17355. All relevant data sets for this study are included on GitHub, https://github.com/baharehsorouri/sphing_climategradient.

## SUPPLEMENTAL MATERIAL

Supplemental material spectrum.00236-24-s0001.pdf Table S1 and Fig. S1 to S4. Supplemental table includes list of core genes and supplemental figures include phylogeny, ordinations, and genome-based trait factor loadings.

ASM does not own the copyrights to Supplemental Material that may be linked to, or accessed through, an article. The authors have granted ASM a non-exclusive, world-wide license to publish the Supplemental Material files. Please contact the corresponding author directly for reuse.
