# Supplementary material for: Sphingomonas clade and functional distribution with simulated climate change
Source: Microbiol Spectr. 2024 Apr 4;12(5):e00236-24. doi: 10.1128/spectrum.00236-24 (PMC11064482; doi:10.1128/spectrum.00236-24)
Supplement: Supplemental material — Table S1 and Fig. S1 to S4. Supplemental table includes list of core genes and supplemental figures include phylogeny, ordinations, and genome-based trait factor loadings. [file spectrum.00236-24-s0001.pdf]

Supplemental Figures and Tables

Supplemental Table 1. List of 23 core genes selected from *Sphingomonas* genomes and appended to Chase et al., (2017) reference database

Protein	Name
L1	rplA
L2	rplB
L3	rplC
L4	rplD
L5	rplE
L6	rplF
L10	rplJ
L11	rplK
L13	rplM
L14	rplN
L15	rplO
L16	rplP
L18	rplR
L24	rplX
S2	rpsB
S3	rpsC
S8	rpsH
S9	rpsI
S11	rpsK
S12	rpsL
S13	rpsM
S17	rpsQ
S19	rpsS

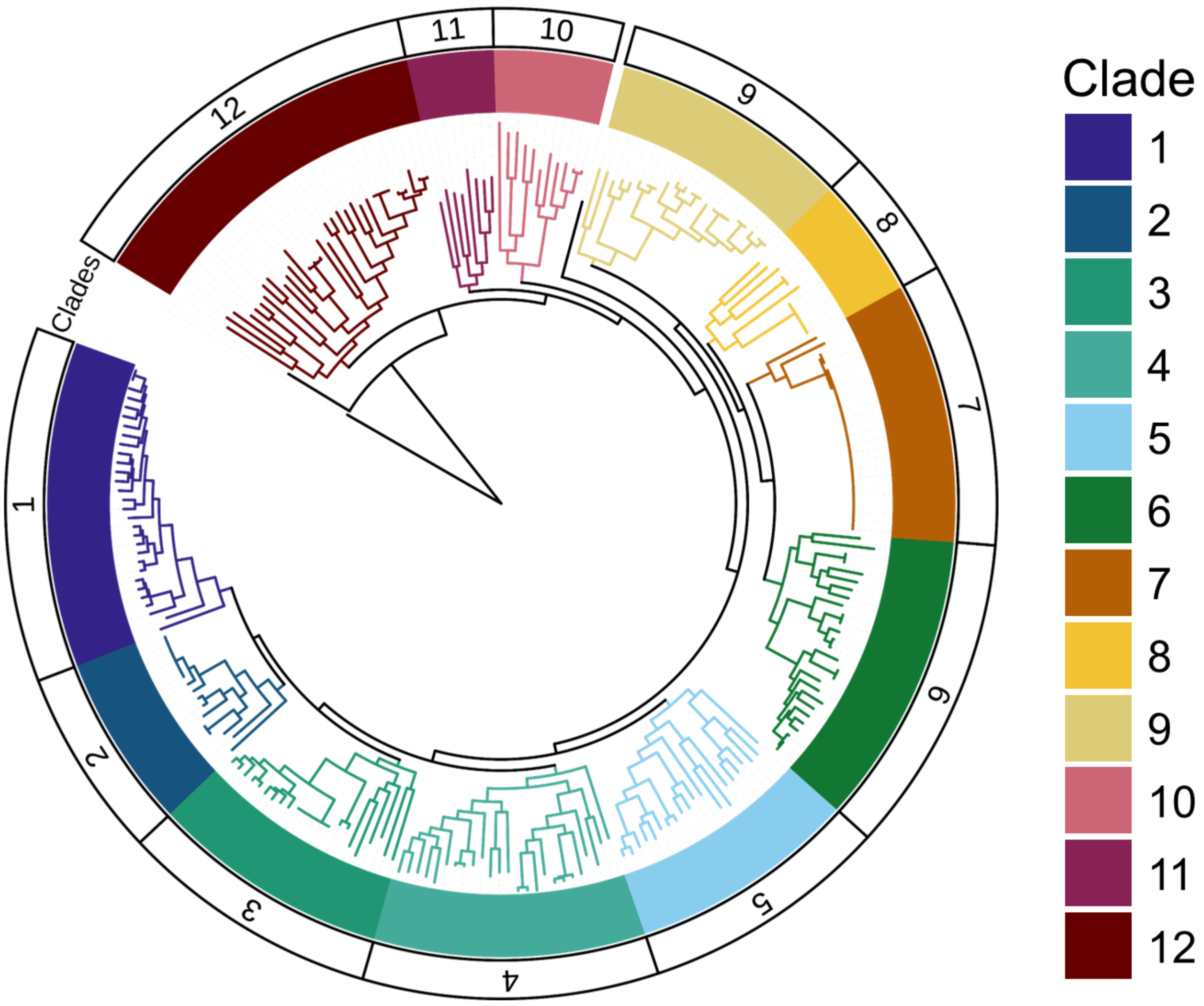

Supplemental Figure 1. Phylogenetic tree of 252 publicly available, high-quality *Sphingomonas* genomes. The tree was built with 404 core genes and separated into 12 clades based on their divergence from a common ancestor. Clades are color coordinated, and the tree is rooted by a *Rhodospirillum centum SW* (Accession: CP000613) outgroup (2). Reproduced from Sorouri et al. 2023 under CC-BY version 4.0.

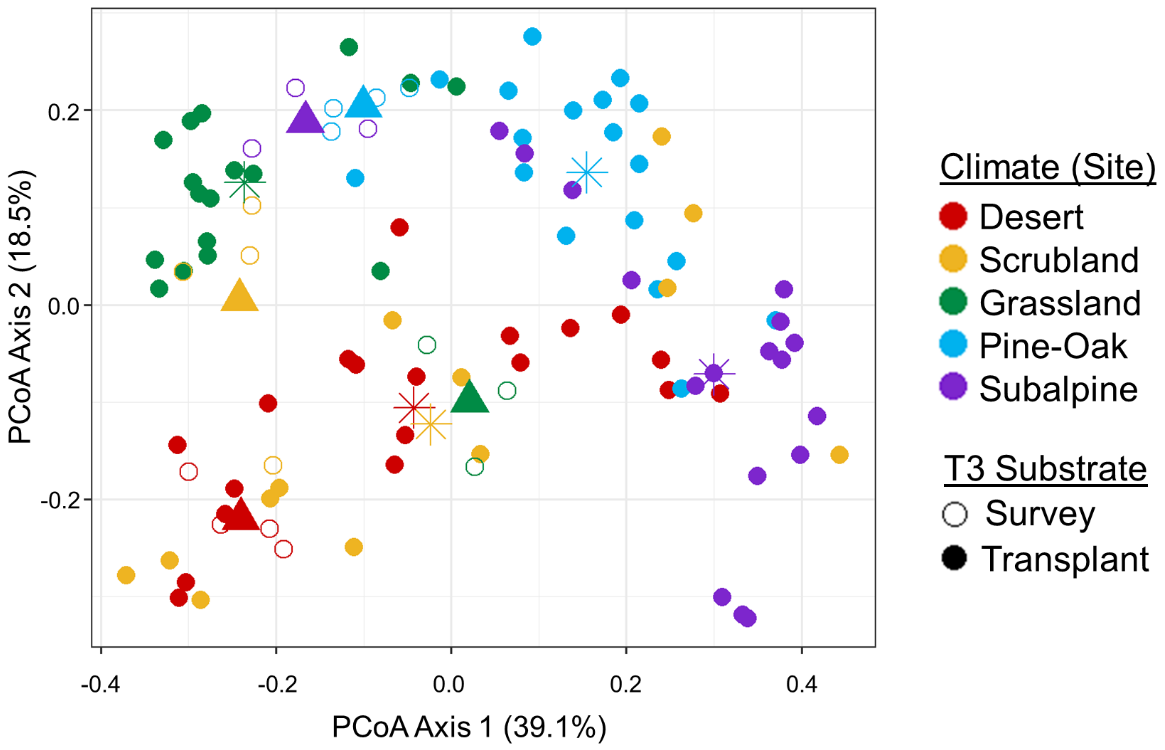

Supplemental Figure 2. Principal coordinate analysis of *Sphingomonas* clade relative abundances within survey and transplant samples after 18 months. The colors reflect the sites, and the ordination was calculated with Bray-Curtis dissimilarity distances. Triangles represent survey centroids and asterisks represent the centroids of transplant samples.

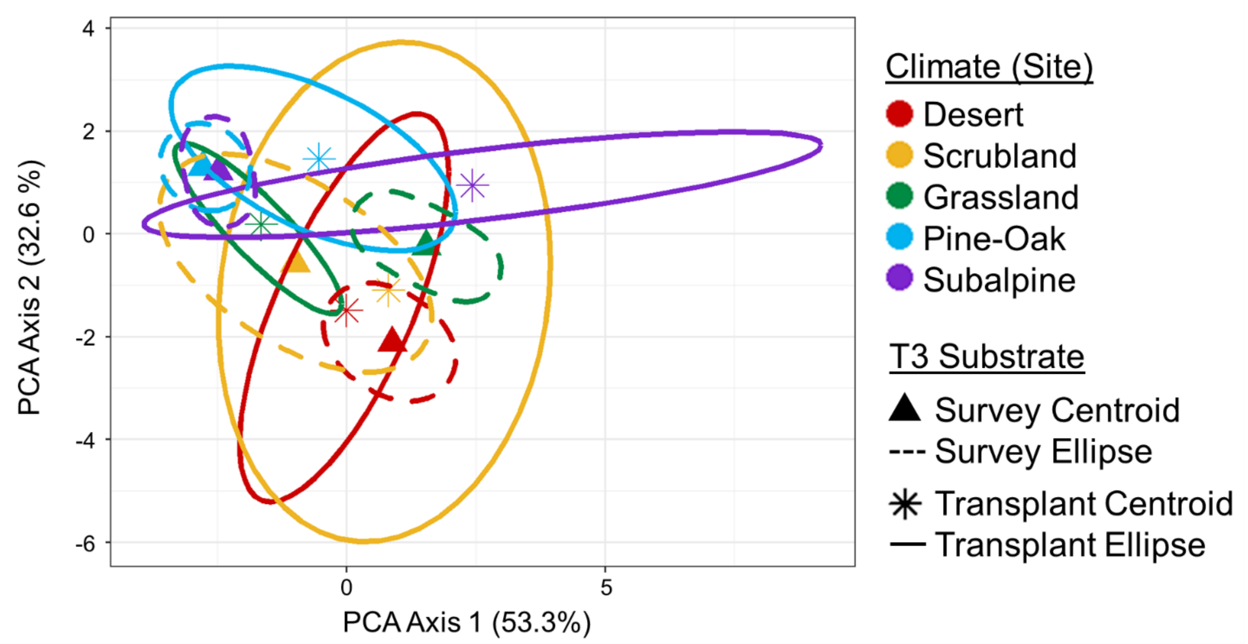

Supplemental Figure 3. Principal component analysis of predicted genome-based functional traits within survey and transplanted samples after 18 months. The colors reflect the site, triangles represent survey centroids, and asterisks represent the centroids of transplant samples. Dashed lined ellipses with a 95% confidence interval encompass survey points, while solid lined ellipses encompass the transplanted points. The subalpine survey does not have an ellipse due to insufficient points following rarefaction. Pseudo-clades were not included in the principal component analysis calculations.

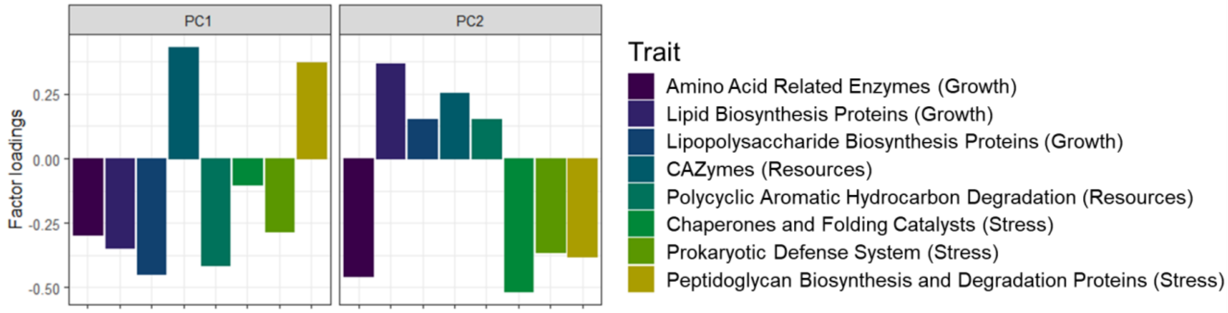

Supplemental Figure 4. ***Sphingomonas*** **genome-based trait factor loadings on the first and second principal components.** Amino acid related enzymes, lipid biosynthesis proteins, and lipopolysaccharide biosynthesis proteins represent the growth yield (Y) life history strategy of the YAS triangle. CAZymes and polycyclic aromatic hydrocarbon degradation proteins reflect resource acquisition (A). Chaperones and folding catalysts, prokaryotic defense system and the peptidoglycan biosynthesis and degradation proteins indicate the stress tolerance (S) life history strategy.

References

1. Chase AB, Karaoz U, Brodie EL, Gomez-Lunar Z, Martiny AC, Martiny JBH. 2017. Microdiversity of an Abundant Terrestrial Bacterium Encompasses Extensive Variation in Ecologically Relevant Traits. MBio 8:e01809-17.

2. Sorouri B, Rodriguez CI, Gaut BS, Allison SD. 2023. Variation in Sphingomonas traits across habitats and phylogenetic clades. Front Microbiol 14:1193.
